# *Lactobacillus johnsonii* and host communication: insight into modulatory mechanisms during health and disease

**DOI:** 10.3389/frmbi.2023.1345330

**Published:** 2024-01-16

**Authors:** Llilian Arzola-Martínez, Keerthikka Ravi, Gary B. Huffnagle, Nicholas W. Lukacs, Wendy Fonseca

**Affiliations:** ^1^ Department of Pathology, University of Michigan, Ann Arbor, MI, United States; ^2^ Mary H. Weiser Food Allergy Center, University of Michigan, Ann Arbor, MI, United States; ^3^ Department of Molecular, Cellular, and Developmental Biology, University of Michigan, Ann Arbor, MI, United States

**Keywords:** *Lactobacillus johnsonii*, gut microbiota, gut-lung axis, probiotics, microbiota metabolites

## Abstract

*Lactobacillus johnsonii* is a commensal bacterium that has been isolated from vaginal and gastrointestinal (GI) tracts of vertebrate hosts, including humans, rodents, swine, and poultry. *Lactobacillus*-based probiotic supplements are popular because of the health advantages they offer. Species such as *L. johnsonii* are particularly interesting due to their potential health-promoting properties. Here, we reviewed the research on specific strains of *L. johnsonii* that have been studied in the context of health and disease and delved into the underlying mechanisms that aid in preserving host homeostasis. The utilization of *L. johnsonii* strains has been widely linked to numerous health benefits in the host. These include pathogen antagonism, control of mucosal and systemic immune responses, reduction of chronic inflammation, modulation of metabolic disorders, and enhanced epithelial barrier. These findings suggest that *L. johnsonii* plays a critical role in maintaining host homeostasis, highlighting its potential as a probiotic.

## 1 Introduction

*Lactobacillus johnsonii* is a Gram-positive, homofermentative, non-spore-forming rod-shaped host-adapted bacterium (Zheng et al., 2020) with lactic acid being its predominant end product from sugar metabolism (Lebeer et al., 2008). Several strains of this species have been isolated from vaginal and gastrointestinal (GI) tracts of vertebrate hosts, including humans, rodents, swine, and poultry (Ravi et al., Pridmore et al., 2004; Leonard et al., 2014; Wu et al., 2016; Guerrero-Preston et al., 2017; Dec et al., 2018; Zhang et al., 2019; Ahire et al., 2021; Reed et al., 2022). The abundance of this bacterium in various niches is often influenced by external factors such as diet, antibiotic treatment, and invading microbes (Mason et al., 2012a; Mason et al., 2012b; Antonissen et al., 2016; Thompson et al., 2023). *L. johnsonii*, like other well-known *Lactobacillus* species, is of particular interest due to its potential health-promoting properties, which mark this specie as a probiotic candidate, defined by the FAO/WHO as ““live microorganisms which, when administered in adequate amounts, confer a health benefit on the host” (Hill et al., 2014).

As a commensal bacterium, *L johnsonii* needs to survive, colonize, multiply and exert its function in the acidic and high bile concentrated conditions in the gut (Stavropoulou and Bezirtzoglou, 2020). For these purposes it has developed resistance and tolerance mechanisms against stressors, while also competing with other indigenous microbes in this niche (O’Flaherty et al., 2018; Zhang et al., 2019; Stavropoulou and Bezirtzoglou, 2020; Bagon et al., 2021). *L. johnsonii* is surrounded by an outer packaged protein shell called S layer. In addition, extracellular peptidoglycan, teichoic acids, and capsular and exo-polysaccharides help to protect and keep cellular integrity and adherence to the host, while the mechanism for stress sensing and export systems complemented the stress resistance machinery (Lebeer et al., 2008). Furthermore, *L. johnsonii* can adapt to the host’s nutritional environment because its genome encodes a high number of the phosphotransferase system (PTS) and ATP-binding cassette (ABC) transporters as well as amino acid protease and peptidases that enable the uptake and utilization of a variety of sugars and amino acids available in the host GI tract microenvironment (Fujisawa et al., 1992; Lebeer et al., 2008; Zhang et al., 2019; Boucard et al., 2022). *In vitro* studies have shown that *L. johnsonii* L531 can produce higher levels of short-chain fatty acid (SCFA) (butyric acid, acetic acid) and lactic acid, having an impact on the metabolic profile and the gut resident microbiota (He et al., 2019). These metabolites are known to promote the maturation of the host immune system and regulate the onset and progression of inflammatory responses (Rooks and Garrett, 2016; Richards et al., 2016).

The inter-strain variations in carbohydrate utilization profile, as well as cell wall composition, determine *L. johnsonii*’s health-promoting and immunomodulatory properties (Fujisawa et al., 1992; Zhang et al., 2019; Schar-Zammaretti and Ubbink, 2003; Guinane et al., 2011). As a result, while *L. johnsonii* is a good probiotic candidate, the different strains of this specie must be independently investigated per the Food and Agriculture Organization of the United Nations (FAO), which guidelines demand to include the source of isolation, characterization, and a credible case presented for their health effects, to be called ‘probiotic’ (Hill et al., 2014).

*L. johnsonii* strains such as NCC 533 (also known as La1) is a commercially available probiotic. Several studies, including *in vitro*, animal models, and clinical trials have shown NCC 533 binding properties to host mucosal cells, as well as its ability to inhibit gut pathogens, stimulate the immune system and metabolic functions, enhance the mucosal barrier and improve human intestinal microbiota (Neeser et al., 2000; Granato et al., 2004; Pridmore et al., 2004; Bergonzelli et al., 2006; Yamano et al., 2006; Inoue et al., 2007; Denou et al., 2008). Several of these health-promoting activities are also observed in other *L. johnsonii* strains when administered to different animal models (La Ragione et al., 2004; Kingma et al., 2011; Fonseca et al., 2017; He et al., 2019; Charlet et al., 2020; Zou et al., 2020). Notably, the survival capacity and safety of *L. johnsonii* strains N6.2 and 456 supplementation have been studied in healthy human volunteers (Marcial et al., 2017; Davoren et al., 2019). *L. johnsonii* strain N6.2 is under clinical trials for its probiotic effect on Type I diabetes (T1D) onset in children, adolescents, and adults (Clinical Trial: NCT03961854, 2019-2023; Clinical Trial: NCT03961347, 2020-2026), while *L. johnsonii* strain MH-68 have shown promising results in the glycemic control and immunomodulation (Wang et al., 2022).

This review summarizes the existing scientific literature on the mechanisms by which *L. johnsonii* affects health and disease progression. Our goal is to comprehend the effects of *L. johnsonii* on various health outcomes.

## 2 *Lactobacillus johnsonii*: impact on health and disease

### 2.1 *L. johnsonii* and the modulation of Gastrointestinal health

Different regions of the GI tract, such as the mouth, stomach, small intestine, and colon, have unique environmental conditions, including variations in pH, nutrients availability, and oxygen levels. These variations created distinct niches for different microorganisms to thrive (Thursby and Juge, 2017). Scientists are studying how gut bacteria affect health and its potential role in treating gastrointestinal disorders (Bidell et al., 2022)*. L. johnsonii* strains as probiotics have been shown to enhance gut health in humans and animals (Marcial et al., 2017; Yang et al., 2022b; Yang et al., 2022c). Microbes can colonize various regions of the GI tract and impact other microbial communities throughout the entire digestive system.

#### 2.1.1 *L. johnsonii* and the Gastrointestinal epithelial barrier

**Figure 1:**
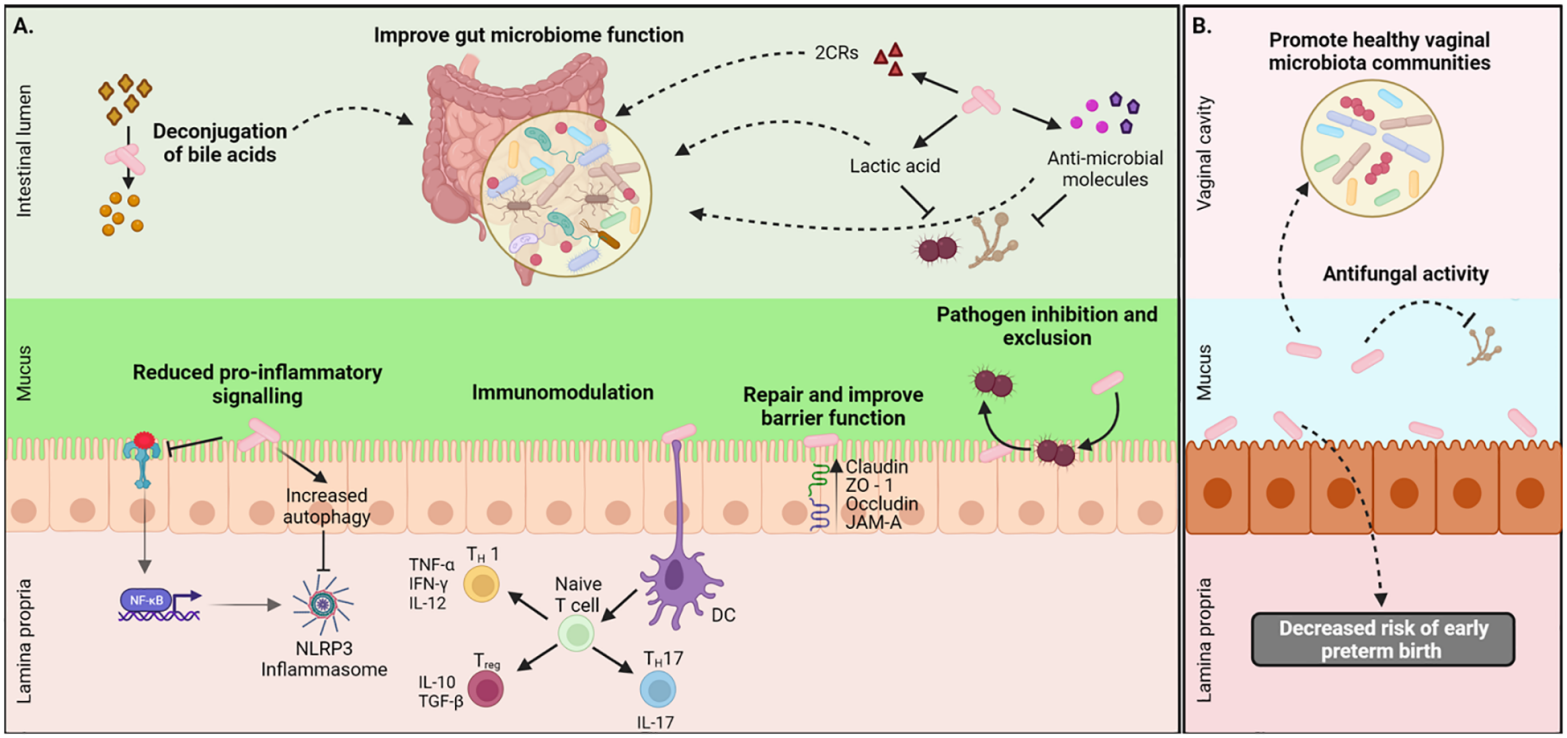
Local health benefits conferred by *L. johnsonii* administration. **(A)** *L. johnsonii* secretes metabolites like lactic acid, hydrogen peroxide, antimicrobial peptides, and bile salt hydrolases (BSH) that facilitate pathogen inhibition and improved gut microbiome function. *L. johnsonii* also inhibits pathogen-induced activation NLRP3 inflammasome via inhibition of TLR4-mediated signaling and promotion of autophagy. It interacts with epithelial cells and repairs barrier function by increasing the expression of tight junction proteins like claudin and occludin. *L. johnsonii* also has immunomodulatory functions. For example, it stimulates dendritic cells (DC), resulting in downstream modulation of both pro-and anti-inflammatory cytokine secretion and thus mediating a Th1/Th2/Treg immune balance response. **(B)** *L. jonhsonii* colonize the vagina of healthy women were display its antifungal properties to promote a healthy vaginal microbiota. Created with BioRender.com.

The intestinal epithelial barrier regulates immunity, nutrient absorption, digestion, and hormone production as well as metabolic processes (Lee et al., 2018). The tight junction (TJ) complex between epithelial cells maintains the intestinal barrier, regulates selective paracellular transit of ions, water, and solutes, and limits the transit of microorganisms, food allergens, and macromolecules (Lynch and Pedersen, 2016; Lee et al., 2018). Several studies have demonstrated the capacity of different *L. johnsonii* strains such as MG, L531, BS15, and 135-1-CHN to enhance the barrier function by upregulating TJ related genes (ZO-1, Occludin, and Claudin-1) (Xin et al., 2014; Liu et al., 2015; Mu et al., 2017; Chen et al., 2021; Lyu et al., 2023), as well as direct interaction with the Junctional Adhesion Molecule-2 (JAM-2) (Bai et al., 2022). Postnatal administration of *L. johnsonii* N6.2 to T1D-prone rats showed no morphological differences between groups in the structure of the villus however, an upregulated expression of claudin-1 and decreased expression of occludin was observed in the *L. johnsonii*-supplemented group, as well as decreased intestinal pro-inflammatory response, showing the ability of *L. johnsonii* N6.2 to ameliorate the intestinal barrier dysfunction (Valladares et al., 2010). The oral administration of *L. johnsonii* promoted the activation of the TLR1/2-STAT3 pathway and increased the number of anti-inflammatory macrophages, leading to IL-10 release and improvement of DSS-induced colitis in mice (Jia et al., 2022). In contrast, clinical studies evaluating the effect *L. johnsonii* NCC 533 supplementation in patients after intestinal resection for Chron’s disease reported that *L. johnsonii* NCC 533 failed to prevent endoscopic recurrence after six months (Marteau et al., 2006; Van Gossum et al., 2007). These studies demonstrated the potential benefits and limitations of *L. johnsonii* in improving intestinal barrier function and reducing epithelial inflammation (**Figure 1**).

#### 2.1.2 *L. johnsonii*: Control of pathogens and regulation of the immune response in the GI

Oral microbiota equilibrium can be affected by inflammatory conditions, such as periodontitis (Manos, 2022). It has been reported that oral pathobiont *Porphyromonas gingivalis* is highly expanded during chronic periodontitis and is associated with several inflammatory disorders, from atherosclerosis to colitis. It plays an important role in establishing and expanding gut pathobionts, highlighting the importance of the oral-gut axis in the development of GI tract pathologies (Kitamoto et al., 2020). *Lactobacillus* bacteria and specifically *L. johnsonii* strains, have been used as an alternative approach to the control of pathobionts associated with periodontitis and dental cavities because of their anti-biofilm activity, which alters the ability of pathobionts to colonize (Jaffar et al., 2016; Giordani et al., 2021). Controlling oral pathogens and oral inflammatory diseases could also impact individuals’ gut microbiota composition and overall health (Imai et al., 2021).

**Figure 2:**
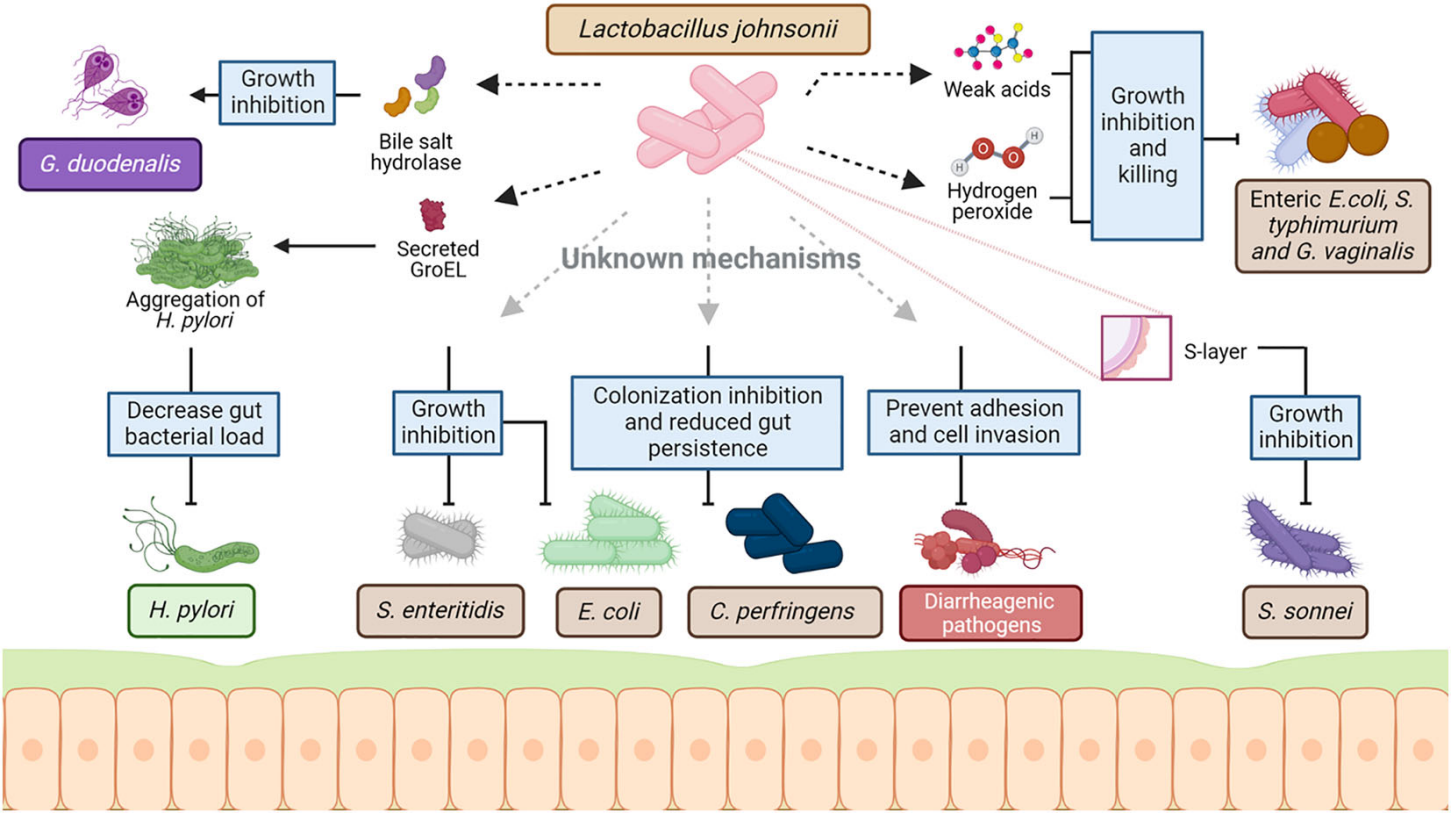
Mechanism of interaction between *L. johnsonii* and commensal and pathogenic bacteria. As a host-associated species, *L. johnsonii* interacts with the resident microbiota as well as invading pathogens to establish stable colonization in the niche. This interaction is multifaceted and involves both secreted and cell surface molecules of *L. johnsonii*. *L. johnsonii* produces bacteriocins and bacteriolysis that target *Lactobacillus* and *Enterococcal* species in a strain-specific manner. Weak acids like lactic acid produced by *L. johnsonii* act together with hydrogen peroxide, inhibiting and killing enteric, vaginosis-associated, and uropathogenic pathogens like pathogenic *E. coli, S. typhimurium* and *Gardnerella vaginalis*. Several cell surface structures of *L. johnsonii* are also involved in antimicrobial activities. GroEL is a surface-associated protein that triggers aggregation of *H. pylori* upon secretion by *L. johnsonii.* This aggregation is hypothesized to cause the rapid exclusion of *H. pylori* from the GI tract upon *L. johnsonii* administration. Similarly, S- layer protein on *L. johnsonii* cell surface can inhibit *S. sonnei* growth. Other cell surface-associated structures like EPS, LTA, EF-t,u, and specific carbohydrate-binding receptors – all involved the in adhesion of *L. johnsonii* to epithelial cells and mucin – are hypothesized to play a role in competing with commensal microbes and pathogens for mucosal binding sites. *L. johnsonii* also inhibits the growth of other pathogens like *S. enteritidis* and reduces gut persistence of *E. coli* and *C. perfringens* through a yet unknown mechanism. Created with BioRender.com.

Extensive research on various *L. johnsonii* strains demonstrates the pathogen-inhibiting property of this bacterium in the GI tract, often via secretion of antimicrobial molecules, lowering the pH of the environment and competing for similar niches. The supplementation of *L. johnsonii* has modulated several intestinal pathogens, such as *Helicobacter pylori*, *Salmonella* spp., pathogenic *Escherichia coli*, and *Clostridium perfringes* (**Figure 2**).

The ability of *L. johnsonii* to inhibit *Helicobacter pylori* infection has been widely studied. Supplementation of *L. johnsonii* in animal models infected with *H. pylori*, resulted in reduced pathogen load, mobility, and aggregation in the gastric mucosa (Sgouras et al., 2005; Isobe et al., 2012; Aiba et al., 2015; Aiba et al., 2019). *L. johnsonii* encode for and secretes a cell surface structure protein named GroEL, which triggers *H. pylori* aggregation under *in vitro* conditions, (Bergonzelli et al., 2006). This interaction could lead to the rapid exclusion of *H. pylori* observed in different *in vivo* studies (Sgouras et al., 2005; Isobe et al., 2012; Aiba et al., 2015; Aiba et al., 2019). A clinical trial using *L. johnsonii* Lj1 fermented milk in *H. pylori*-positive volunteers showed reduced antral gastritis, inflammatory score in the gastric mucosa, and decreased density of *H. pylori (* *Pantoflickova et al., 2003* *).* In contrast, oral supplementation with *L. johnsonii* NCC 533 supernatants did not control *H. pylori* persistence in humans (Michetti et al., 1999). However, heat killed/lyophilized as well as viable *L. johnsonii* No.1088. were shown to reduce gastrin-mediated acid production, by decreasing the number of gastrin-positive cells in mice stomach (Aiba et al., 2015) and its combination with anti-*H. pylori* urease immunoglobulin Y (IgY) significantly reduced *H. pylori* infection(Aiba et al., 2019). More studies are needed to determine the necessity of viable bacteria to report a positive effect of *L johnsonii* in *H. pylori* treatment.

In addition to pathogen exclusion, *L. johnsonii* supplemented mice resulted in reduced *H. pylori*-related inflammation by diminished gastric mucosa inflammatory leukocyte (neutrophils, lymphocytes, macrophages) infiltration and proinflammatory chemokine and cytokine expression (macrophage inflammatory protein 2, keratinocyte-derived cytokine) (Sgouras et al., 2005). Additional *in vitro* studies showed that the incubation of *H. pylori*-infected human adenocarcinoma *AGS* cell lines with *L. johnsonii* NCC 533 cultures supernatants reduced the expression of *H. pylori*-induced IL-8, without affecting the bacterial viability (Sgouras et al., 2005). These studies showed the immunomodulatory effect of *L. johnsonii* in the control of *H. pylori*-related inflammation.

Current therapies for *H. pylori* infection include antimicrobial agents and inhibitors of gastric acid secretion, such as proton pump inhibitors (PPI) and vonoprazan. In a mouse model, these drugs decreased the population ratio of *L. johnsonii (* *Nadatani et al., 2019* *).* Interestingly, *L. johnsonii* supplementation in a model of indomethacin-induced small intestinal damage in combination with PPI or vonopazan, protects mice from intestinal injury (Nadatani et al., 2019). These data illustrate the distinct characteristics of *L. johnsonii* and its potential used as part of therapeutic protocols to alleviate the adverse effects of medications and synergistically reduce detrimental bacterial growth and tissue inflammation.

Different studies suggest that *L. johnsonii* L531 has the potential to control other intestinal pathogens, such as *Salmonella* sp. (He et al., 2019; Xia et al., 2020; Yang et al., 2020; Chen et al., 2021; Yang et al., 2022b). Oral supplementation with *L. johnsonii* L531 to newly weaned piglets, one week before challenged with *Salmonella enteric* serovar Infantis, reduced diarrhea severity, intestinal inflammation, and tissue damage. The modulation of the inflammatory response led to epithelial protection and reduced abundance of *Salmonella* in the ileum mucosa (He et al., 2019; Yang et al., 2022b). The protective effects of *L. johnsonii* on *Salmonella* sp. immunopathogenesis, have been associated with the inhibition of the NOD pathway, the modulation of endoplasmic reticulum stress, and the promotion of autophagy degradation (Yang et al., 2020; Yang et al., 2022b); as well as the regulation of NLRC4 and NLRP3 inflammasome, proinflammatory cytokines expression via NFκB signaling and inhibition of mitochondrial damage (Xia et al., 2020; Chen et al., 2021).

**Figure 3:**
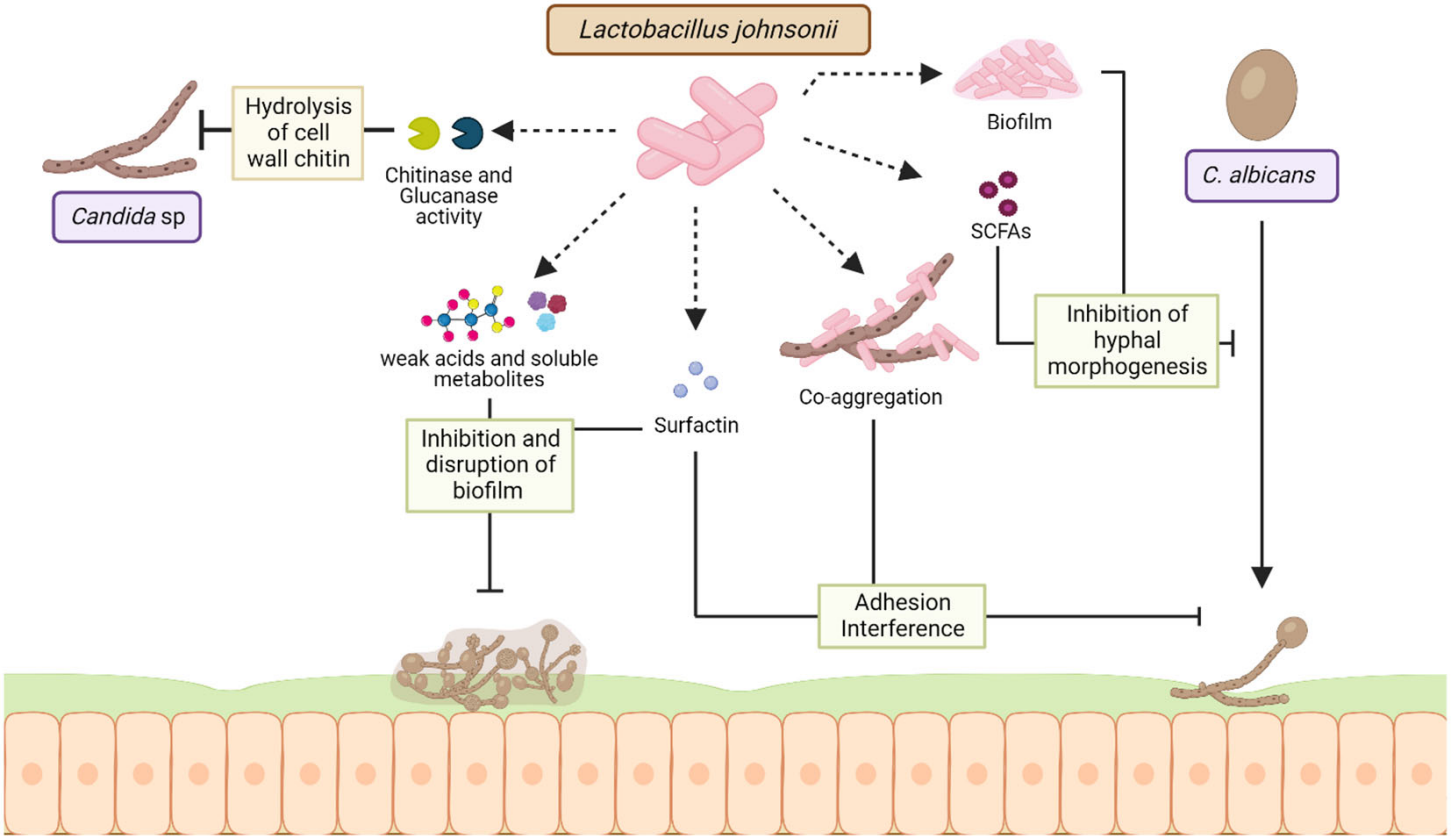
Mechanisms of interaction between *L. johnsonii* and *Candida* sp. *L. johnsonii* antagonizes the growth of *C. albicans* both *in vitro* and in the GI tract via secreted and cell surface molecules. Chitinase and glucanase-like hydrolytic enzymes secreted by *L. johnsonii* can degrade the fungal cell wall, causing rapid decreases in *Candida* viability during co-culture. Acidification of the niche due to weak acids and other soluble metabolites produced by *L. johnsonii* can inhibit the formation of *Candida* biofilms and disrupt established *C. albicans* biofilm structure. Strains of *L. johnsonii* also encode the surfactin gene, a biosurfactant that prevents biofilm formation and inhibits *C. albicans* adhesion. Additionally, *L. johnsonii* biofilm structure and production of SCFAs like butyric acid can inhibit *C. albicans* hyphal morphogenesis, thereby affecting its pathogenicity. Finally, *L. johnsonii* and *C. albicans* co-aggregate *in vitro*, a characteristic hypothesized to interfere with adherence and trigger rapid exclusion. Created with BioRender.com.

*In silico* studies identified three potential gene products in *L. johnsonii* NCC 533 genome that may catalyze the known antimicrobial factor hydrogen peroxide (H_2_O_2_) synthesis. *L. johnsonii* NCC 533 and other *L. johnsonii* strains produced H_2_O_2,_ which is hypothesized to play a role in the elimination of *Salmonella enterica* serovar Typhimurium SL1344 *in vitro (* *Pridmore et al., 2008* *)*. Additionally, it has been suggested that H_2_O_2_ and lactic acid produced by *L. johnsonii* act co-operatively to kill enteric, vaginosis-associated, and uropathogenic pathogens, such as enteric pathogenic *E. coli, S. typhimurium* and *Gardnerella vaginalis* (Atassi and Servin, 2010). Acidification of the microenvironment is an anti-microbial mechanism employed by several lactic acid bacteria (LAB). Lactic acid and other weak acids produced by lactobacilli have been known to exhibit pathogen-inhibitory function by reducing the pH in the surrounding environment (Peter, 1993; Servin, 2004). Interestingly, *L. johnsonii* NCC 533 inhibits *Salmonella enterica* serovar Typhimurium SL1344 growth only at a low pH of 4.5, but not at pH 6.5 (Fayol-Messaoudi et al., 2005) (**Figure 3**).

*L. johnsonii* NCC 533 has been shown to control pathogens by producing bile-salt-hydrolase (BSH) (Travers et al., 2016). This enzyme hydrolyzes the amino bonds of conjugated bile salts to generate deconjugated bile salts (cholic, deoxycholic, and chenodeoxycholic acids) (Begley et al., 2006; Travers et al., 2016). It has been shown that BSH might play a role in antiparasitic activity against *Giardia* sp., a protozoan intestinal parasite that causes giardiasis, by inhibiting the proliferation of *Giardia* sp. trophozoites (Travers et al., 2016; Allain et al., 2017). The BSH present in the supernatants of *L. johnsonii* NCC 533 prevent *Giardia* sp. growth *in vitro* by converting bile’s non-toxic components into highly toxic components to *Giardia* sp. (Travers et al., 2016). Furthermore, mice treated with recombinant BSH during *Giardia duodenalis* infection presented decreased numbers of trophozoites in the small intestine, showing the antiparasitic effect of the BSH-L enzyme and suggesting that the mechanism by which *L. johnsonii* controls intestinal parasite infection is through the production of specific metabolic enzymes (Allain et al., 2017).

*L. johnsonii* can prevent the adhesion and cell invasion of several diarrheagenic bacteria, including enteropathogenic *E. coli* (EPEC), enterotoxigenic *E. coli* (ETEC), *Yersinia pseudotuberculosis* and *Salmonella typhimurium*, to intestinal epithelial cells (Bernet et al., 1994; Liu et al., 2015). This broad inhibitory effect of *L. johnsonii* strains was initially attributed to the non-specific steric interference of receptors needed for pathogen colonization. However, there is evidence suggesting the involvement of a more direct inhibitory mechanism by a recent work of Zang et al., which showed that the S-layer protein of *L. johnsonii* F0421 inhibited *Shigella sonnei* adhesion to HT-29 cells (Zhang et al., 2012). Thus, *L. johnsonii* strains can regulate the colonization of intestinal pathogens by controlling their adherence to the mucosal epithelium.

*L. johnsonii* has also been shown to provide protection against *Citrobacter rodentium*-induced colitis in an animal model by modulating the innate immune signaling pathways, as well as inflammatory responses and ER stress (Zhang et al., 2021). *L. johnsonii* administration in abiotic mice did not abrogate *Campylobacter* sp. jejune growth but reduced the expression of pro-inflammatory cytokines (such as IL-6, MCP1, and TNF) in the intestinal tract (Bereswill et al., 2017). *L. johnsonii* NJ3 supplementation of mice infected with enterohemorrhagic *E. coli* increased the diversity of the intestinal microbiota and improve the diarrhea index, body weight, and liver index (Hu et al., 2021)*.In vitro* studies have shown that *L. johnsonii* L531 inhibit NLRP3 activity by promoting autophagy leading to reduced *Escherichia coli*-induced cell damage (Zou et al., 2020).

In the last decade, the number of antibiotic-resistant pathogenic bacteria and the search for alternative therapies to help control bacterial infections have increased. Probiotics, as well as fecal transplantation from healthy individuals, is an alternative therapy for the treatment of antibiotic-resistant bacteria and for re-establishing healthy gut microbiota in individuals with chronic diseases (Reyman et al., 2022). Studies by Ekmekciu et al. compared the efficacy of fecal microbiota transplantation (FMT) from healthy mice to oral supplementation with *L. johnsonii* in mice subjected to broad-spectrum antibiotic treatment for eight weeks. The antibiotic treatment diminished immune cell populations in the intestine, mesenteric lymph nodes, and spleen. In contrast, after antibiotic treatment, FMT and *L. johnsonii* supplementation increased CD4+, CD8+, and regulatory T cells (Tregs) cells in the small intestine and the spleen. Treatment *with L. johnsonii* also maintains colonic IL-10 production (Ekmekciu et al., 2017). This study showed the potential of *L. johnsonii* supplementation in individuals with dysbiosis caused by antibiotic treatment and its use as a therapeutic intervention for bacterial infection with an antibiotic-resistant phenotype.

Gut microbiota dysbiosis can exacerbate intestinal fungal infections, and *Candida* sp. is the most frequent cause of yeast infection (Charlet et al., 2020; Jawhara, 2022). *L. johnsonii* and *Bacteroides thetaiotaomicron* interact with *Candida* sp. and promote fungal cell wall degradation via chitinase-like and mannosidase-like activity, inhibiting fungal growth (Charlet et al., 2020). It has been shown that the administration of these two bacterial during DSS-induced colitis controlled the growth of pathogenic *E. coli*, *Enterococcus faecalis*, and *Candida glabrata* in the intestine, intestinal inflammation by downregulating intestinal IL-1β, TLR9, and NF-κB activation and upregulating IL-10 (Charlet et al., 2020). In a different approach, Bertolini et al. observed that changes in the microbial composition and function induced by dietary sucrose generated an increased abundance of *Lactobacillus* sp. and decreased *Candida albicans* burden in a murine model of oropharyngeal candidiasis during immunosuppression (Bertolini et al., 2021). The same authors showed that *L. johnsonii* MT-LB4 has an inhibitory effect on *Enterococcus faecalis* and planktonic *Candida albicans* growth *in vitro (* *Bertolini et al., 2021* *)*. Furthermore, the production of oleic acid and palmitic acid by *L. johnsonii* during interaction with colonic epithelial cells has been associated with anti-inflammatory and antifungal properties in a DSS- induced colitis mice model (Charlet et al., 2022). Studies have shown that *L. johnsonii* MT4 exhibited pH-dependent and pH-independent antagonistic interactions with *C. albicans*, by inhibiting its growth and biofilm formation via nutrient competition and the production of metabolites with anticandidal activity with a similar sequence to antifungal compounds, such as Bacillomycin D, Surfactin, glucanase, and Msp1/p75 (Vazquez-Munoz et al., 2022)*. L. johnsonii* JCM1022 inhibits *C. albicans* hyphal morphogenesis *in vitro*, via butyric acid production (Tang et al., 2010). Therefore, *L. johnsonii* in the GI tract can control the growth of fungal pathogens and hinder biofilm formation through *L. johnsonii*-derived metabolites, as well as anti-inflammatory and anti-fungal properties (**Figure 3**).

### 2.2 *L. johnsonii* and autoimmune diseases

Dysbiosis of the gut microbiota has been hypothesized to promote autoimmune disorders, such as type 1 diabetes (TD1) (Chagwedera et al., 2019). T1D results from the destruction of insulin-producing β cells via autoreactive T cells, which affects the self-regulation of blood sugar in the body. Notably, T and B-cell-deficient rodents fail to develop T1D, even when carrying predisposing genetic mutations (Christianson et al., 1993). Evidence shows that the resident gut microbiota is involved in the progression of T1D and that altering the gut microbiota using probiotics can be a therapeutic tool to help manage T1D (Vaarala et al., 2008; Dovi et al., 2022).

It has been observed that rats which spontaneously develop TD1 due to genetic predisposition (BioBreeding diabetes-prone rats -BBDP), have increased susceptibility to infections (Roesch et al., 2009). One of the differences between BBDP rats and BioBreeding diabetes-resistant rats (BBDR) is their gut microbiota, specifically, *Lactobacillus* and *Bifidobacterium* abundance, which are dominant bacterial communities that negatively correlated with the onset of T1D (Lai et al., 2009; Roesch et al., 2009; Valladares et al., 2010). Interestingly, oral administration of *L. johnsonii* N6.2 to BBDP rats, decreased the incidence of diabetes by altering intestinal microbiota, decreasing the host intestinal oxidative stress response, and modifying the intestinal pro-inflammatory response, while *Lactobacillus reuteri* fails to mediate the resistance to T1D (Valladares et al., 2010). This was further accompanied with changes in dendritic cell phenotype that contributed to the Th17 lymphocyte’s immune polarization in mesenteric lymph nodes and spleen (Lau et al., 2011).In addition, it was described that *L. johnsonii* N6.2 derived lipids promoted a tolerogenic-migratory DC-like phenotype that could enhance regulatory T cells responses and prevent the initiation of the autoimmune process (Cuaycal et al., 2023). TLR9 activation seems to be implicated in this polarizing-tolerogenic mechanism (Kingma et al., 2011). In addition, to reshape the Treg/Th17 commitment, *L. johnsonii* N6.2 can modulate the assembly of the inflammasome, evidenced by lower levels of mature caspase-1 in BBDP rats (Teixeira et al., 2018). Immunoregulatory properties of *L. johnsonii* N6.2 derived H_2_O_2_ abolished the activity of the rate-limiting enzyme for tryptophan catabolism, indoleamine 2,3-dioxygenase (IDO) known by its capacity to induce the proinflammatory cytokine IFNγ (Valladares et al., 2013). A pilot clinical study with this strain supports the safety and tolerance of *L. johnsonii* N6.2 administration in healthy humans’ patients (Marcial et al., 2017). However, few clinical studies have supported the benefits of probiotic supplementation in patients with T1D (Dovi et al., 2022). In a clinical study, patients diagnosed with T1D (onset age 6 to 18 years old) were supplemented daily for 60 days with placebo or a capsule containing active probiotics including *L. johnsonii* MH-68. The probiotics mix had a positive impact on glycemic and glycated hemoglobin levels in the blood, increased the presence of beneficial bacteria species, such as *Bifidobacterium animalis*, *Akkermansia muciniphila* and *Lactobacillus salivarius* and reduced inflammatory cytokines in the serum of patients with TD1. Glycemic control and immunomodulation persisted 3 months after stopped probiotics intake (Wang et al., 2022). Although probiotics cannot cure T1D, they can help manage symptoms and be used as a supportive treatment for T1D and other autoimmune diseases.

**Figure 4:**
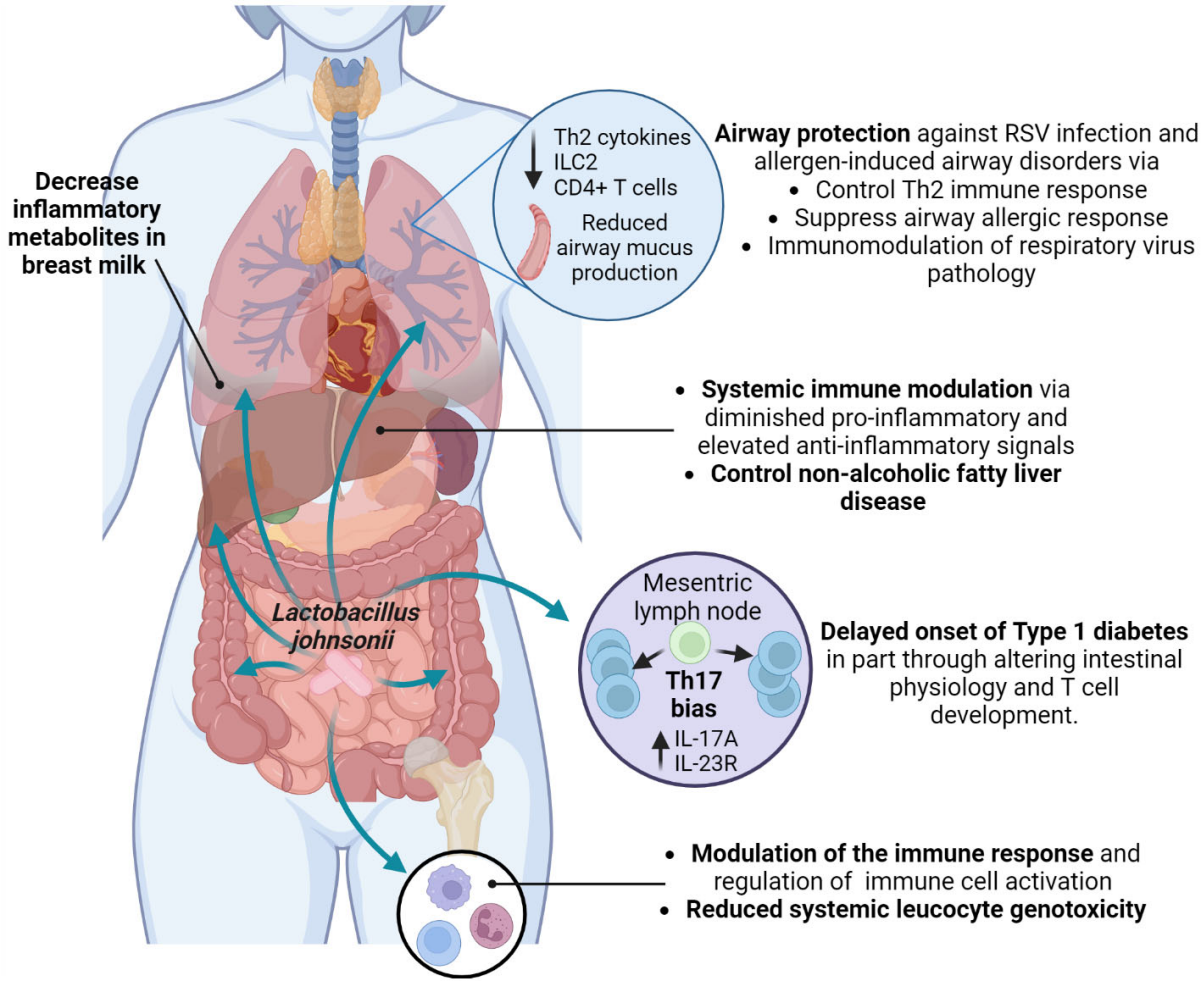
Systemic health benefits conferred by *L. johnsonii* administration. *L. johnsonii* immunomodulatory properties are related to its capacity to alter microbiota composition and function, changing the bacterial communities’ metabolites profile. In addition, *L. johnsonii* express and produce substances and release vesicles with local and systemic anti-inflammatory and metabolic effects that modulate susceptibility to Th2 (allergic) responses and RSV infection in the airways and impacted onset and clinical manifestations of autoimmune and metabolic diseases. Created with BioRender.com.

Furthermore, it has been suggested that *L. johnsonii* can release bioactive molecules with immunomodulatory effects (Harrison et al., 2021). Microbial extracellular vesicles have been reported in feces, blood, and urine and show different patterns depending on the individual’s health status. There is an increasing interest in studying these microbial extracellular vesicles as possible biomarkers for disease assessment and as immunomodulators of disease over the use of live organism (Park et al., 2021; Diez-Sainz et al., 2022; Yang et al., 2022a). *L. johnsonii* N6.2-derived nanovesicles (NV10) are rich in glycerophosphoglycerols and contain several unique and differentially expressed proteins compared to the bacteria cellular membrane (Harrison et al., 2021). *L. johnsonii* N6.2 extracellular vesicles could upregulate IL-10 expression in macrophages, promoting the M2 tolerogenic phenotype through STAT3 activation, while in the human pancreatic cell line Blox5, it reduced cytokine-induced apoptosis (Teixeira et al., 2022). *L. johnsonii* N6.2 derived phospholipids modified bone marrow-derived dendritic cells (BMDCs) transcriptional signature, triggering the expression of anti-inflammatory cytokine Il10 (Cuaycal et al., 2023), suggesting that *L. johnsonii* N6.2 nanovesicles’ phospholipids components might have an immunomodulatory function. Interestingly, human pancreatic islets treated *in vitro* with *L. johnsonii* N6.2 extracellular vesicles showed significant upregulation of the expression of glucose transporter Solute Carrier Family 2, Member 6 (*SLC2A6)*, also known as glucose transporter 6 (GLUT6), suggesting that *L. johnsonii* can induce glucose uptake by pancreatic islets under high glucose conditions, and increase insulin secretion (Teixeira et al., 2022). These studies showed possible mechanisms by which *L. johnsonii* generated changes at a location distant to the gut via extracellular vesicle and possible mechanisms of *L. johnsonii* N6.2 to attenuate the onset of T1D by immunoregulation (**Figure 4**).

It is important to investigate the effects of *L. johnsonii*-derived extracellular vesicles and live *L. johnsonii* to understand the different outcomes generated among them and the specific role of the extracellular vesicles in the modulation of inflammatory responses and autoimmune diseases.

### 2.3 *L. johnsonii* and metabolic diseases

Metabolic diseases due to poor diets and obesity also cause significant disease manifestations. Diet can affect gut microbiota composition and function, as well as metabolic processes that can lead to the development of metabolic syndrome, cardiovascular disease, and type 2 diabetes (Ginsberg and MacCallum, 2009; O’Toole and Shiels, 2020; Wicinski et al., 2020). Recent studies have evaluated the effect of *L. johnsonii* N6.2 supplementation in a high-fat diet (HFD) rat model to induce metabolic syndrome. The authors observed that *L. johnsonii* N6.2 in combination with phytophenols reduced mTORC1-activating phosphorylation of AKT and other genes expression downstream mTORC1 signaling pathway in HFD-fed females (Kling et al., 2018). mTOR and AKT functions are associated with glucose and lipid metabolism, which are involved in metabolic syndrome (Saxton and Sabatini, 2017), suggesting that *L. johnsonii* N6.2 supplementation may help to diminish fat deposition and could modulate the development of the metabolic syndrome.

The close relationship between the gut microbiome and obesity has been extensively studied (Wicinski et al., 2020). An elevated prevalence of obesity worldwide is associated with increased non-alcoholic fatty liver disease (NAFLD) (Wong and Ahmed, 2014). A study by Jinge et al. investigated the effect of *L. johnsonii* BS15 administration on the development of NAFLD in obese male mice. The authors observed that *L. johnsonii* BS15 supplementation protected mice from hepatic steatosis and hepatocyte apoptosis when exposed to a HFD. The protective effect was attributed to enhanced liver antioxidative defense, as well as inhibition of insulin resistance and decreased expression of acetyl-CoA carboxylase 1, fatty acid synthase, and peroxisome proliferator-activated receptor γ. Long-term alterations were observed in the gut microbiota of obese mice, with an increased abundance of *Lactobacillus* sp. and specifically *L. johnsonii* after 63 days of supplementation. After 119 days of probiotic supplementation with *L. johnsonii*, obese mice showed decreased serum LPS levels, and reduced intestinal permeability and pro-inflammatory response by downregulating TNFα expression (Xin et al., 2014). These studies confirm the crucial role of the microbiome in maintaining metabolic homeostasis in the host and reducing associated illnesses, as well as the long-term effect of *L. johnsonii* supplementation in the gut microbiota composition and functionof host metabolism and inflammatory status (**Figure 4**).

### 2.4 *L. johnsonii* and the Reproductive system

The vaginal microbiome is a dynamic ecosystem influenced by external or environmental stressors (sexual activity and personal hygiene) and intrinsic physiological conditions, such as hormonal changes, sexual development, pregnancy, and disease states. *Lactobacillus* sp. is the most abundant microorganism in the vaginal bacterial community and is a well-known pH acidifier (Greenbaum et al., 2019). Although recent data suggest that bacterial vaginosis (BV) results from polymicrobial disruption of the vaginal microbiota, the alkaline pH in BV patients has been related to decreased lactic acid production by *Lactobacillus* sp. (Greenbaum et al., 2019). One of the *Lactobacillus* specie that colonized the vagina and intestine of healthy women is *L. Johnsonii (* *Dobrut et al., 2018* *)*. *L. johnsonii* UBLJ01, isolated from the vagina of healthy women, was found to inhibit the growth of *Gardnerella vaginalis*, *Proteus mirabilis*, and *Candida albicans (* *Ahire et al., 2021* *)*. The therapeutic effects of *L. johnsonii* B-2178 and *Lactobacillus acidophilus* were tested in a rat model of vulvovaginal candidiasis and observed that both lactobacilli reduced *C. albicans* vaginal load and hyphae formation and significantly reduced proinflammatory cytokines IL-17 and IFNγ. Interestingly, only *L. johnsonii* B-2178 protected the vaginal mucosa epithelium from histopathological changes (Elfeky et al., 2023), suggesting that the presence of *L. johnsonii* in the reproductive tract may help to control the growth of pathogens and maintain a healthy environment (**Figure 1**).

### 2.5 *L. johnsonii* and the perinatal and infant health

Clinical studies during the prenatal, perinatal, and infant periods are relevant since infancy is a critical period when the human microbiome starts to establish, and alterations in the microbiome composition during early life can impact overall host homeostasis and promote the development of disease risk factors. In the last decade, there has been increased interest in studying the short- and long-term effects of pre- and post-natal microbiome alterations in mothers and newborns (Fujimura et al., 2016; Fonseca et al., 2017; Fonseca et al., 2021). The study of maternal and infant microbiomes is an opportunity to explore the critical role of *L. johnsonii* in physiological outcomes during pregnancy and the infant’s health.

Supplementing with probiotics during pregnancy can alter the composition of the gut and vaginal microbiota, breastmilk microbes, impact mother and infant immunity, and types of molecules that can be passed to the newborn (Rautava et al., 2012; Kuang and Jiang, 2020; Fonseca et al., 2021; Lehtoranta et al., 2022). A study evaluating the effect of prenatal supplementation with *L. johnsonii* MR1 in mice observed changes in the gut microbiota and the systemic metabolic profile of supplemented mothers and their offspring. Offspring from *L. johnsonii*-supplemented mothers showed an expansion of bacteria belonging to *Lachnospiraceae* and *Muribaculaceae* families, similar to *L. johnsonii*-supplemented mothers. In addition, the systemic metabolic profile of mothers and offspring, as well as the mother’s breastmilk metabolic profile, displayed similarity in the decreased presence of inflammatory metabolites (9,10-dihydroxyoctadecenoic acid (DiHOME), linoleic acid metabolite, and guanosine) (Fonseca et al., 2021). Similar metabolite changes were found in clinical studies with birth cohorts and showed that increases in systemic metabolites, such as DiHOME were associated with severe allergic disease in children (Fujimura et al., 2016). Likewise, clinical studies have shown that prenatal probiotic supplementation prevents infection, preterm delivery during pregnancy, and the manifestation of GI disorders and allergic responses in newborns (Baldassarre et al., 2018; Navarro-Tapia et al., 2020) (**Figure 4**).

To our knowledge, there are no clinical studies evaluating the effects of *L. johnsonii* administration during pregnancy. However, the presence of *Lactobacillus gasseri/Lactobacillus johnsonii* in the vagina of pregnant women has been associated with a decreased risk of early preterm birth (Tabatabaei et al., 2019). These data from animal models and clinical studies emphasize the potential role of *L. johnsonii* in women’s reproductive health, including controlling pathogens and promoting healthy pregnancies. Additionally, early-life *L. johnsonii* exposure may be critical in establishing a healthy microbiome. Thus, the study of prenatal *L. johnsonii* supplementation represents an opportunity to assess the potential benefits in mothers and infants. Testing its use prenatally in mothers with vaginal dysbiosis and postnatally in infants born via C-section could be especially interesting.

### 2.6 *L. johnsonii* and the Respiratory system: gut-lung axis

The gut-lung axis concept postulates that alterations in the gut microbiota affect lung homeostasis. A correlation between the composition of the gut and lung microbiota from birth to adulthood suggests an interconnection. Altering the gut microbiome affects lung immunity and microbiota composition (Markey et al., 2018; Yagi et al., 2022). This could also be an effect generated by systemic microbiome-derived metabolites or even the previously described extracellular vesicles.

Exposure to environmental factors impacts the gut microbiome composition and has been associated with increased risk of asthma development (Fujimura et al., 2016; Yagi et al., 2022). Early-life exposure to livestock or pets significantly diversifies the gut microbiome and reduces allergy and asthma risk, highlighting the link between environment and microbiome composition and function (Ownby et al., 2002; von Mutius and Vercelli, 2010). House dust from dog owners was found to confer protection against ovalbumin and cockroach allergen-induced airway diseases when orally administered to mice (Fujimura et al., 2014). Notably, this protection in mice models was associated with an increased abundance of *L. johnsonii* MR1 in the gut (Fujimura et al., 2014; Ravi et al., 2023). Mice supplemented with *L. johnsonii* MR1 before an airway-allergen or respiratory syncytial virus (RSV) challenge presented reduced Th2-airway-related immune response and reduced mucus deposition in the airways (Fujimura et al., 2014; Fonseca et al., 2017). This effect was related to an attenuated proinflammatory phenotype in dendritic cells and increased pulmonary Treg cells due to altered systemic metabolic profile (Fujimura et al., 2014; Fonseca et al., 2017). Furthermore, maternal *L. johnsonii* MR1 supplementation protected the neonates from severe RSV immunopathology, presenting a significant decrease in airway mucus deposition, Th2 cytokines production, as well as reduced numbers of innate lymphocyte cells 2 (ILC2) and CD4+ T cells in the lung. Furthermore, offspring born from *L. johnsonii*-supplemented mothers maintain the immunomodulatory effect until adulthood. Adult offspring were infected with RSV and showed reduced RSV immunopathology, suggesting that prenatal *L. johnsonii* supplementation impacts mother and offspring gut microbiome composition and function and metabolic profiles that might alter long-term the mucosal and systemic immune response (Fonseca et al., 2021). This study emphasizes the importance of the mother’s microbiome and the transfer of gut microbiota and immune-modulatory metabolites from mother to offspring to control allergic disease and respiratory pathogens during infancy (Fonseca et al., 2021). Pre- and post-natal probiotics have been recommended for patients with a high risk of developing allergic diseases (Fiocchi et al., 2015). Overall, these studies emphasize the importance of the gut microbiota (gut-lung axis) in maintaining respiratory health by delivering metabolites, regulating metabolism, improving immune system maturation, and possibly lung development. *L. johnsonii* may improve lung health and modulate the immune response to pathogens. Clinical studies are needed to assess its potential use in controlling inflammation in the respiratory tract (**Figure 4**).

### 2.7 *L. johnsonii* and skin barrier

Similar to the gut, skin microorganisms play an essential role in educating the cutaneous innate and adaptive immune response, and skin microbiota dysbiosis has been associated with skin diseases (Byrd et al., 2018), suggesting that manipulation of skin microbiota could help control skin pathologies, such as atopic dermatitis (AD) and eczema. Interestingly, reshaping of the gut microbiota, metabolic functions, and immune responses by oral probiotic interventions has been proposed to positively impact the clinical manifestations of inflammatory skin disorders such as AD (Fang et al., 2021). However, AD patients have skin dysbiosis characterized by a high prevalence of *Staphylococcus aureus (* *Brussow, 2016* *)* and a lower presence of *Lactobacillus* species in the skin, as well as increased abundance of *Clostridium difficile* and bifidobacterial species in the gut (Melli et al., 2020). A connection between the gut microbiome and the skin microorganism community has been suggested, which could potentially impact the immune response of patients who have inflammatory skin conditions.

The benefits of altering skin microbiota by directly applying pre-and probiotics have been reviewed previously (Al-Ghazzewi and Tester, 2014). The microbe-microbe interactions and immunological action of a topical lotion containing heat-treated *L. johnsonii* NCC 533 were assessed in an *in vitro* reconstructed human epidermis (RHE) model. Non-replicative *L. johnsonii* NCC 533 reduced *Staphylococcus aureus* colonization and boosted cutaneous innate immunity by inducing the expression of antimicrobial peptides, such as cathelicidin and β-defensin (Rosignoli et al., 2018). In addition, the topical use of heat-killed *L. johnsonii* NCC 533 in 21 patients with AD and swab positive for *Staphylococcus aureus*, reduced *S. aureus* load and the AD overall score in an open-label, multicenter clinical study (Blanchet-Rethore et al., 2017). These studies showed an alternative use of *L. johnsonii* to control skin pathogens and boost the skin innate immune response. The authors pointed out the importance of non-replicating bacteria in this interaction with the host and argued that heat-killed *L. johnsonii* NCC 533 maintains its ability to stimulate cytokine production and induce the expression of antimicrobial peptides. It is possible that heat-killed *L. johnsonii* activates innate immune receptors by interacting directly with the skin epithelial cells in a TLR2-dependent but TLR4/MD-2-independent manner (Elson et al., 2007), helping to control *Staphylococcus aureus* growth. It is important to note that this intervention is not considered probiotic-mediated, as it does not contain live *L. johnsonii.* The interconnected nature of skin and gut microbiome interactions has not been thoroughly examined; however, they likely interact through their influence on local and systemic immune responses. Additional research is needed to better understand the potential skin health benefits of *L. johnsonii*, offering a valuable research opportunity.

### 2.8 *L. johnsonii* anticarcinogenic activity

Recent findings have highlighted the importance of probiotics for cancer treatment (Slizewska et al., 2021). The gut microbiome’s composition and function are linked to clinical response to immunotherapy for antitumor treatment (Weersma et al., 2020). Furthermore, a reproducible shift in bacterial richness and metabolic pathways has been consistently identified across different cohorts of individuals with colorectal cancer, which opens the possibility of using microbial signatures as biomarkers for intestinal cancer (Thomas et al., 2019). Microbiome-derived metabolites, such as short-chain fatty acids (SCFA), decreased inflammation and cancer cell proliferation (Ocadiz-Ruiz et al., 2017), and regulate the onset and progression of inflammatory responses (Richards et al., 2016). *L. johnsonii* is essential for influencing intestinal microbiota composition and metabolic activity, producing compounds with anticarcinogenic activity, stimulating the immune system, and modulating cell proliferation and apoptosis (Slizewska et al., 2021). Interestingly, *in vitro* and *in vivo* studies have shown that *L. johnsonii* L531 can produce high levels of SCFA, such as butyric, acetic, and lactic acids, affecting the metabolic profile and gut resident microbiota (He et al., 2019). Additionally, a comprehensive analysis of operational taxonomic units (OTU) in a mouse model of ataxia-telangiectasia, a genetic disorder associated with B cell lymphoma, showed that the less cancer-prone mouse colony had higher *L. johnsonii* colonization. Short-term restorative oral treatment with *L. johnsonii* RS-1 decreased systemic genotoxicity and inflammatory state in mice prone to developing cancer by diminishing hepatic T and NK cells, pro-inflammatory cytokines IL-1β and IFN-β levels, and elevated anti-inflammatory cytokines TGF-β and IL-10 (Yamamoto et al., 2013). This study shows the capacity of *L. johnsonii* strains to regulate the inflammatory response in cancer, like other beneficial bacteria that decrease inflammation and cancer cell proliferation and possibly modulate the efficacy of anticancer therapy (Lee et al., 2021). However, the mechanism by which each probiotic intervention exerts its anticarcinogenic activity must be clarified.

## 3 Concluding remarks

*L. johnsonii* is a commensal bacterium that has been isolated from vaginal and gastrointestinal (GI) tracts of vertebrate hosts, including humans, rodents, swine, and poultry. *Lactobacillus*-based probiotic supplements are popular because of the health advantages they offer and species such as *L. johnsonii* are of particular interest due to their potential health-promoting properties. *L. johnsonii* possesses exceptional properties that help it to maintain homeostasis in the host by controlling the expansion of pathogens, modulating metabolic pathways, and regulating the immune response systemically and locally. The modulation and restoration of healthy microbiota by *L. johnsonii* offer positive outcomes and represent an important tool to aid treatments and control specific pathologies’ development by directly modulating microbiota composition and function and, consequently, local and systemic immune responses. While several of these health-beneficial properties have been investigated *in vitro* settings and animal models (**Table 1**), there is still insufficient scientific evidence in humans to support these claims (**Table 2**). Studying the microbiomes of pregnant women and their infants presents an opportunity to investigate the significant role of *L. johnsonii* in impacting physiological outcomes and infant health. Hence, to validate the efficiency of *L. johnsonii* as a therapeutic probiotic, it is necessary to conduct more randomized clinical trials that encompass diverse populations, including individuals of different sexes, ages, and dietary habits. Other important parameters to consider include health status, underlying diseases or conditions, dosage, route and frequency of administration, the location of the study, and ensuring an adequate sample size for accuracy (Dronkers et al., 2020).

**Table 1:** *Lactobacillus johnsonii* studies in animal models and *in vitro* experiments.

Ref.	Model	Strain(s)	Principal Outcome(s)
(Ahire et al., 2021)	*In vitro*	*L. johnsonii* UBLJ01	*L. johnsonii* formed biofilms *in vitro* and had a standard antibiotics susceptibility. Secreted exopolysaccharided and inhibited pathogens growth (*E. coli, Gardnerella vaginalis, Proteus mirabilis*, and *C. albicans*).
(Sgouras et al., 2005; Bergonzelli et al., 2006)	*In vitro* and *H. pylori* infected C57BL/6 mice model	*L. johnsonii* NCC 533	GroE protein facilitated *L. johnsonii* NCC 533 binding to epithelial cells and mucus proteins in a pH-dependent manner and aided *H. pylori* aggregation. *H. pylori* induces pH-dependent IL-8 secretion. *In vivo* studies showed that *L. johnsonii* NCC 533 administration attenuated *H. pylori-*associated gastritis by reducing proinflammatory chemokine, cytokine expression, and immune cell infiltration. *H. pylori*-induced IL-8 secretion is reduced *in vitro* in the presence of neutralized *L. johnsonii* NCC 533 culture supernatants, without loss of *H. pylori* viability.
(Aiba et al., 2015) (Aiba et al., 2019)	Human gut microbiota-associated mice model and germ free mice model	*L. johnsonii* No.1088	*L. johnsonii* No.1088 suppressed gastric acid production and inhibited the growth of *Helicobacter pylori*.
(Yang et al., 2022c)	Chronic diarrhea in rhesus macaques (RMs. *Macaca mulatta*)	*L. johnsonii*	RMs with chronic diarrhea showed a microbiome depleted in *L. johnsonii*, *L. reuteri* and *L. amylovorus*. *L. johnsonii* isolated from asymptomatic RMs possessed probiotic genes encoding lactate dehydrogenases, mucus-binding proteins, bile salt hydrolase and bile salt transporter.
(Mu et al., 2017)	MRL/lpr mice (lupus nephritis model).	Mix of 5 *Lactobacillus* strains including *L. johnsonii* 135-1-CHN	Lactobacillales supplementation had a sex-dependent anti-inflammatory effect. It restored the gut mucosal epithelial barrier, diminished IL-6, upregulated IL-10 and IgG2 levels, and skewed the Treg-Th17 balance in the kidney towards Treg, leading to immunosuppression
(Xin et al., 2014)	High fat diet (HFD) mice model	*L. johnsonii* BS15	*L. johnsonii* BS15 protected mice from hepatic steatosis and hepatocyte apoptosis, enhanced the liver antioxidant defense system, and increased the expression of the fasting-induced adipose factor. *L. johnsonii* BS15 administration modulated gut barrier function and gut microbiota, as well as downregulated TNFα expression in the liver.
(Isobe et al., 2012)	*Helicobacter pylori* infection model of Mongolian gerbil	*L. johnsonii* NCC 533	*L. johnsonii* NCC 533 impaired *Helicobacter pylori* colonization and ameliorated gastritis.
(Nadatani et al., 2019)	Mice Indomethacin (IND)-induced intestinal damage.	*L. johnsonii*	*L. johnsonii* administration protected from IND-induced intestinal damage and reduced IL-1β expression.
(He et al., 2019) (Yang et al., 2020) (Yang et al., 2022b) (Xia et al., 2020) (Chen et al., 2021)	Piglets model of *Salmonella* sp. infection and *in vitro* studies	*L. johnsonii* L531	Supplemented piglets had reduced diarrhea severity, restored tight junctions (ZO-1, Occludin, and Claudin-1), exhibited *Salmonella* sp. clearance, and restored SCFA. Attenuated tissue damage and inflammation and contributed to the maintenance of intestinal homeostasis by reducing expression of pro-inflammatory innate cytokines (IL-6, IL-1β, IL-8, and TNFα) and NOD-related proteins (NOD1/2, RIP2), regulating NLRC4 and NLRP3 inflammasomes assembly and NF-κB signaling pathway (TLR4, MyD88, p-IκBα, and p-p65), reduced ER stress and cellular damage, as well as inhibition of mitochondrial damage and mitophagy, and modulating autophagy degradation.
(Liu et al., 2015)	*In vitro* studies with IPEC-J2 cells	*L. johnsonii* P47-HY	*L. johnsonii* P47-HY supplementation improves the integrity of the gut barrier by stimulating the production of cytoprotective heat shock proteins and fortified cellular defense against enterotoxigenic *Escherichia coli* by regulating tight junction proteins and direct interactions with pathogens.
(Zhang et al., 2012)	*In vitro* studies with HT-29 cells	*L. johnsonii* F0421	*L. johnsonii* F0421 inhibits adherence of *Shigella sonnei* in a dose dependent manner. S-layer proteins on *L. johnsonii* F0421 have a role in this exclusion adhesion process.
(Bereswill et al., 2017; Zhang et al., 2021)	Mice model *of Campylobacter jejuni* infection	*L. johnsonii*	Prophylactic supplementation of *L. johnsonii* did not alter *Campylobacter jejuni* growth, but diminished colonic apoptosis and attenuates colonic hyperplasia, as well as reduced systemic proinflammatory mediators (IL-6, MCP1, TNFα and nitric oxide) and immune cell infiltration in the colonic tissue *L. johnsonii* restored abnormal expression of antimicrobial peptides (lysozyme) and abrogated ER stress–related cell apoptosis.
(Hu et al., 2021)	Mice model *of E. coli* infection	*L. johnsonii* NJ13	Ameliorate the diarrhea index and increased body weight. Improved microbiota structure (reduction of *Helicobacter pylori* and *Shigella*) and increasing in butyric acid-producing bacteria and *Lactobacillus*.
(Ekmekciu et al., 2017)	Secondary abiotic mice	*L. johnsonii*	*L. johnsonii* recolonization increased CD4+ and CD8+ T cells populations in the small intestine and spleen, and sustained IL-10 production in the colon. A minor increased of the frequency of intestinal regulatory and memory/effector T cells and activated dendritic cells was observed.
(Travers et al., 2016; Allain et al., 2017)	Mice model of *Giardia duodenalis* infection *in vitro* studies	*L. johnsonii* NCC 533	*L. johnsonii* La1 genome possessed probiotic genes encoding *bile-salt-hydrolase* (*bsh*) enzymes. BHS enzymes identified in the supernatants of *L. johnsonii* La1 prevent *Giardia duodenalis* growth *in vitro*.
(Zhang et al., 2021) (Jia et al., 2022) (Charlet et al., 2020) (Charlet et al., 2022)	Mice model of colitis	*L. johnsonii*	*L. johnsonii* supplementation alleviated induced colitis in different mice models. *Citrobacter rodentium* -induced colitis model: *L. johnsonii* pretreatment regulated inflammation by diminishing systemic proinflammatory cytokines (TNFα, IL1β, IL6, IL17a, IFNγ and MCP1) and immune cells infiltration (T cells and macrophages) in the gut. Restored concentrations of antimicrobial peptides such as lysozyme and attenuates ER stress-related cell death. DSS-induced colitis model: *L. johnsonii* supplementation alleviated the severity of diarrhea, altered gut microbiota composition by increasing the presence of SCFA-producing bacteria, as well as bacteria with anti-inflammatory, immunomodulatory and antifungal properties.
(Bertolini et al., 2021)	Mice model of fungal infection *in vitro* coculture model	*L. johnsonii* MT-LB4	*C. albicans* infection in immunocompromised mice was associated with enterococci relative abundance. *Lactobacillus* sp. depletion with antibiotics showed a negative correlation between these bacteria genera and the opportunistic bacteria *Enterococcus* in *Candida*-infected mice. *L. johnsonii* has an inhibitory effect on *Enterococcus faecalis* and planktonic *Candida albicans* growth.
(Vazquez-Munoz et al., 2022)	*In vitro*: Coculture of *L. johnsonii* and *C. albicans*	*L. johnsonii* MT4	*L. johnsonii* MT4 has genes encoding products with anticandidal properties (bacteriocin, hydrolases, biosurfactant). *L. johnsonii* MT4 reduced the metabolic activity of *C. albicans* biofilms in a dose–response pattern and impacted its *Candida* dimorphic transition.
(Roesch et al., 2009) (Lai et al., 2009) (Lau et al., 2011; Teixeira et al., 2018) (Valladares et al., 2010) (Valladares et al., 2013) (Kingma et al., 2011)	Bio-Breeding diabetes-prone (BBDP) Rats and non-obese diabetic (NOD) mice	*L. johnsonii* N6.2	*L. johnsonii* bacteria abundance in stools samples differs between diabetes-prone and diabetes-resistant rats. Two cinnamoyl esterases enzymes isolated from *L. johnsonii* N6.2 have potential to mitigates diabetes symptoms. Supplementation with *L. johnsonii* N6.2 isolated from Bio-Breeding diabetes-resistant (BBDR) rats, delays the onset of TD1 in BBDP rats. *L. johnsonii* N6.2 supplementation in BBDP rats pulsed dendritic cells to mediate Th17 bias and modulates the assembly of the inflammasome. H_2_O_2_ produced by *L. johnsonii* N6.2 abolished the rate-limiting enzyme of tryptophan catabolism, indoleamine 2,3-dioxygenase (IDO).
(Harrison et al., 2021) (Teixeira et al., 2022)	*In vitro*	*L. johnsonii* N6.2	*L. johnsonii* N6.2-derived nanovesicles are rich in glycerophosphoglycerols and contains several unique and differentially expressed proteins compared to the bacteria cellular membrane. IgA and IgG antibodies against protein domains from nanovesicles were generated in the plasma of individuals supplemented with *L. johnsonii* N6.2. Nanovesicle-derived bioactive molecules suppressed cytokine-induced apoptosis and promoted a tolerogenic immune environment by skewing macrophages to a M2 tolerogenic phenotype associated to STAT3 activation, expression of AHR-dependent genes, and IL-10 secretion.
(Cuaycal et al., 2023)	*In vitro*	*L. johnsonii* N6.2	Bone marrow-derived dendritic cells (BMDCs) showed an upregulation of maturation-migratory and immunoregulatory related genes when incubated with *L. johnsonii* N6.2 purified phospholipids. These BMDCs presented a tolerogenic-migratory DC-like phenotype, suggesting its capacity to induce a regulatory T cell response.
(Kling et al., 2018)	Rat model of obesity (HFD)	*L. johnsonii* N6.2	*L. johnsonii* N6.2 reduced AKT phosphorylation and downregulated various genes that are part of the downstream signaling pathway of mTORC1 in female rats.
(Elfeky et al., 2023)	Vulvovaginal candidiasis rat model	*L. johnsonii* B-2178	*L. johnsonii* B-2178 reduced *C. albicans* vaginal load and hyphae formation, as well as pro-inflammatory cytokines IL-17 and IFN-γ and NF-κB, while minimized the epithelium damage and restored normal vaginal architecture.
(Fonseca et al., 2017) (Fonseca et al., 2021) (Fujimura et al., 2014)	Mice model of asthma and Respiratory Syncytial Virus (RSV) infection. Neonatal mice model of RSV infection. *In vitro* studies.	*L. johnsonii* MR1	Intestinal *L. johnsonii* MR1 presence was linked with allergic, and RSV reduce immunopathology in mice exposed to house-dust from homes with pets. *L. johnsonii* MR1 oral supplementation to adult mice altered gut microbiome communities and systemic metabolic profile that reduced RSV immunopathology, airway Th2 inflammatory response, and dendritic cell function, as well as increased pulmonary Treg cells. Prenatal supplementation with *L. johnsonii* MR1changed the gut microbiota and the systemic metabolic profile of supplemented mothers and their offspring. *L. johnsonii*-supplemented Mothers and their Offspring showed expansion of Lachnospiraceae families as well as, changes in the systemic and breastmilk’s metabolic profile, that presented reduced levels of inflammatory metabolites. The neonates born from supplemented mother showed reduced RSV immunopathology and dampened Th2 immune response.
(Rosignoli et al., 2018)	*In vitro* human epidermis (RHE) model	Heat-treated *L. johnsonii* NCC 533	Heat-treated *L. johnsonii* suspensions reduced the binding of *Staphylococcus aureus*. Heat-treated *L. johnsonii* induced the presence of antimicrobial peptides.
(Yamamoto et al., 2013)	Mice model of Ataxia-telangiectasia	*L. johnsonii* RS-1	*L. johnsonii* restoration diminished genotoxicity by reducing hepatic NK and T cells, pro-inflammatory cytokines IL-1β and IFN-β and increasing expression of anti-inflammatory cytokines TGF-β and IL-10.
(Hsieh et al., 2012)	*In vitro* and rat model	*L. johnsonii* MH-68	*L. johnsonii* MH-68 suppressed *H. pylori* urease activity, dampened its adhesion capacity to epithelial cells and inhibits bacteria growth *in vitro*. *L. johnsonii* MH-68 supplementation effectively decreased *H. pylori* load in the gastric mucosa and lowered the expression of IL-8 and lymphocyte infiltration.

APCs ,antigen presenting cells; AHR, aryl hydrocarbon receptor; BSH, bile-salt-hydrolase ; BBDP, Bio-Breeding diabetes-prone; BBDR, Bio-Breeding diabetes-resistant; CFU, colony formation units; DSS, dextran Sulfate Sodium; DHA, docosahexanoic acid; ER , endoplasmic reticulum ; IND, indomethacin; KC, keratinocyte-derived cytokine; HFD, high fat diet; MIP-2, macrophage inflammatory protein 2; mTORC1, mTOR complex 1; MOI, multiplicity of infection ; NK, natural killer cells; NLRC4, NLR family apoptosis inhibitory protein CARD domain-containing protein 4; NSAID, NLRP3, non-steroidal anti-inflammatory drugs; nucleotide-Binding Domain, Leucine-Rich–Containing Family, Pyrin Domain–Containing-3; OA, oleic acid; PA, palmitic acid; PUFAs, polyunsaturated fatty acids; RSV, respiratory syncytial virus; RM, rhesus macaques; SCFA, short chain fatty acids; SLE, systemic lupus erythematosus; TCR, T cells receptor; TD1, type 1 diabetes.

**Table 2:** *Lactobacillus johnsonii* studies in human cohorts.

Ref.	Study design	Strain(s)	Principal Outcome(s)
(Davoren et al., 2019)	Healthy humans (11) with normal diet	Daily 100mL of yogurt containing 10^10^ CFU of *L. johnsonii* 456 for 7 days	Daily consumption as part of yogurt for 7 days impacted the microbiota composition, elevating the presence of lactic acid bacteria and *L. johnsonii* 456 DNA unique sequences were still detected in human fecal samples weeks after intake was stopped.
(Marcial et al., 2017)	Randomized, double-blind, placebo-controlled trial	*L. johnsonii* N6.2 (5 × 10^8^ CFU per capsule) during 8 weeks with 4 weeks washout period Placebo: skim milk	*L. johnsonii* N6.2 impacted the innate and adaptative immune systems and effects on the tryptophan metabolism are dependent on the baseline microbiota composition, specifically the lactic acid bacteria population.
(Wang et al., 2022)	Randomized, double-blind placebo-controlled trial	Probiotic mix including *L. johnsonii* MH-68 Placebo: insulin therapy without probiotic mix	Probiotics mix changed the microbiota composition of TD1 patients, increasing *Bifidobacterium animalis, Akkermansia muciniphila*, and *Lactobacillus salivarius*, reduced fasting blood glucose levels and serum proinflammatory cytokines.
(Marteau et al., 2006; Van Gossum et al., 2007)	Multicenter, randomized, controlled trial/Randomized, double blind, placebo-controlled trial	*L. johnsonii* NCC 533 (10^9-10^ CFU)	Supplementation failed to prevent early endoscopic recurrence after post-ileocecal resection of macroscopic lesions in patients with CD.
(Michetti et al., 1999; Pantoflickova et al., 2003)	Randomized, double-blind study/Randomized, double-blind, placebo-controlled trial	*L. johnsonii* LJ1/ *L. johnsonii* NCC 533 supernatant	*L. johnsonii* LJ1 reduced *H. pylori-associated* gastritis, *H. pylori* load, and increased mucus production. *L. johnsonii* NCC 533 supernatant inhibited *H. pylori* growth *in vitro*, but not *in vivo*
(Tabatabaei et al., 2019)	Nested case-control study (94 women with spontaneous preterm birth cases)	*Lactobacillus gasseri/Lactobacillus jonhsonii*	*Lactobacillus gasseri/Lactobacillus jonhsonii* oligotype was associated with a decreased risk of early spontaneous preterm birth.
(Blanchet-Rethore et al., 2017)	Open-label, multicenter clinical study	Heat-treated *L. johnsonii* NCC 533, non-replicating probiotic. Lotion	Application of *L. johnsonii* NCC 533 lotion in patients with atopic dermatitis, reduced Staphylococcus aureus colonization as well as atopic dermatitis lesions.

CFU, colony forming units; CD, Crohn disease; TD1, Type 1 diabetes.

These trials should also adhere to intent-to-treat principles, conduct prospective evaluation, and use an adequate control group (Evans, 2010; Lim and In, 2019) to generate scientific evidence of the mechanism of action of *L. johnsonii* and validate its benefit during health and disease.

## Author contributions

LA: Writing – original draft, Writing – review & editing. KR: Writing – original draft, Writing – review & editing. GH: Funding acquisition, Writing – original draft, Writing – review & editing. NL: Funding acquisition, Writing – original draft, Writing – review & editing. WF: Writing – original draft, Writing – review & editing.
